# Methamphetamine and Inflammatory Cytokines Increase Neuronal Na^+^/K^+^-ATPase Isoform 3: Relevance for HIV Associated Neurocognitive Disorders

**DOI:** 10.1371/journal.pone.0037604

**Published:** 2012-05-25

**Authors:** Gurudutt Pendyala, James L. Buescher, Howard S. Fox

**Affiliations:** Department of Pharmacology and Experimental Neuroscience, University of Nebraska Medical Center, Omaha, Nebraska, United States of America; Kaohsiung Chang Gung Memorial Hospital, Taiwan

## Abstract

Methamphetamine (METH) abuse in conjunction with human immunodeficiency virus (HIV) exacerbates neuropathogenesis and accelerates neurocognitive impairments in the central nervous system (CNS), collectively termed HIV Associated Neurocognitive Disorders (HAND). Since both HIV and METH have been implicated in altering the synaptic architecture, this study focused on investigating alterations in synaptic proteins. Employing a quantitative proteomics approach on synaptosomes isolated from the caudate nucleus from two groups of rhesus monkeys chronically infected with simian immunodeficiency virus (SIV) differing by one regimen, METH treatment, we identified the neuron specific Na^+^/K^+^-ATPase alpha 1 isoform 3 (ATP1A3) to be up regulated after METH treatment, and validated its up regulation by METH *in vitro*. Further studies on signaling mechanisms revealed that the activation of ATP1A3 involves the extracellular regulated kinase (ERK) pathway. Given its function in maintaining ionic gradients and emerging role as a signaling molecule, changes in ATP1A3 yields insights into the mechanisms associated with HAND and interactions with drugs of abuse.

## Introduction

The neurological, motor and cognitive impairments in AIDS collectively termed HIV Associated Neurocognitive Disorders (HAND) or neuroAIDS encompasses a broad range of neurological abnormalities including asymptomatic neurocognitive impairment; HIV associated mild cognitive motor disorder and the most severe disease, HIV associated dementia [1], [2]. The increasing usage of drugs of abuse, in particular methamphetamine (METH) abuse poses a significant co-morbid factor in HIV-infection due to the mutual association with risky behaviors such as intravenous injection [3] and risky sexual behavior with multiple partners [4], [5]. Clinical studies have documented evidence that HIV positive individuals dependent on METH have a higher rate of neuropsychological impairment than those that do not use METH or METH users that are not HIV infected [6]. This overlap in the HIV infected and METH using population raises the potential importance of an interaction between these agents that affects the brain. While the neurotoxicity resulting from HIV infection involves toxic viral proteins or inflammatory mediators produced by infected and/or activated macrophages and activated glial cells [7], [8] METH amplifies this activation cascade [9], [10] as well as directly affecting the neurons [11].

Although several advances have been made in identifying molecules that have provided valuable insights into mechanisms of neuropathogenesis, only minimal information exits on synaptic changes associated with HIV/METH induced neurodegeneration. Synapses are key structures involved in neurotransmission and neuroplasticity. Both HIV and METH lead to alterations in the synaptic architecture thus leading to neurodegeneration [12]. In order to investigate differential patterns in synaptic proteins, we isolated synaptosomes, which represent isolated synapses containing the pre and post synaptic components, from the caudate nucleus from rhesus monkeys chronically infected with SIV and differing by one regimen, METH treatment. The caudate was chosen as it is a target of both HIV and METH. Quantitative proteomics identified the neuron specific Na^+^/K^+^-ATPase alpha 3 isoform 3 (ATP1A3) to be up regulated after METH treatment amongst a panel of synaptic proteins showing differential expression. Studies were then performed to ascertain the role of ATP1A3 in HIV/METH induced CNS dysfunction.

## Materials and Methods

### Ethics statement

Materials used in these studies were from animal work performed under IACUC approval (from the Scripps Research Institute, 07-0067). Animal welfare was maintained by following NIH (Public Health Service, Office of Laboratory Animal Welfare) and USDA guidelines by trained veterinary staff and researchers under Association for Assessment and Accreditation of Laboratory Animal Care certification, insuring standards for housing, health care, nutrition, environmental enrichment and psychological well-being. These met or exceeded those set forth in the *Guide for the Care and User of Laboratory Animals* from the National Research Council of the US National Academy of Sciences. All efforts were made to ameliorate suffering of the animals, including the use of anesthesia with ketamine, xylazine, and phenobarbital at necropsy.

### Rhesus macaques, SIV infection and METH treatment

Full details of the METH treatment and course of infection have been described in our previous publication [13]. Briefly six rhesus monkeys (*Macaca mulatta*), free of type D simian retroviruses, Cercopithecine Herpesvirus 1, simian T-cell leukemia virus type 1, and SIV, were infected with an *in vivo* serial passage derivative of SIVmac251 [14], [15]. At 19 weeks of infection, animals were matched for viral load, and three were treated with an escalating dose regimen of METH injected intramuscularly (5 week ramp-up followed by 18 week maintenance at 25 mg/kg/week); the other three animals received PBS injections on the same schedule. All animals were sacrificed at 42 weeks post infection, before the development of AIDS, and tissues harvested following PBS perfusion under lethal anesthesia.

### Isolation of Synaptosomes

Synaptosomes were isolated using a discontinuous sucrose density gradient by differential centrifugation using a standard isolation procedure [16]. 250 mg of the caudate nucleus from each animal was homogenized in 10 volumes of ice-cold buffered sucrose (0.32 M sucrose, 5 mM HEPES containing complete EDTA-free protease inhibitor cocktail (Roche Applied Science, Indianapolis, IN) with twelve strokes in a dounce homogenizer using a Wheaton overhead stirrer at 250 rpm. The homogenate was centrifuged at 1,000× g for 10 min at 4°C to yield the post nuclear supernatant. The post nuclear supernatant was centrifuged at 12,000× g for 20 min at 4°C to yield the crude synaptosomal pellet. The crude synaptosomal pellet was gently dissolved in the buffered sucrose and layered over the discontinuous sucrose gradient comprising of 0.6, 0.8 and 1.2 M sucrose from top to bottom and centrifuged at 145, 000× g for 90 min at 4°C. The purified synaptosomes were isolated from the interface of 0.8 and 1.2 M layers, diluted with 10 volumes of buffered sucrose and washed at 145, 000× g for 30 min at 4°C. The pellet was carefully resuspended in the buffered sucrose and protein concentration was determined using BCA kit (Pierce, Pittsburg, PA) with BSA as a standard.

### iTRAQ labeling

Isobaric Tag for Relative and Absolute Quantitation (iTRAQ) labeling was performed as described in our earlier studies [17], [18]. A pooled sample was made of the synaptosomal fractions from all samples. 100 µg protein from each individual sample and the pool were digested with trypsin and labeled separately using the iTRAQ (Applied Biosystems, Foster City, CA) standard protocols for the 8-plex kit. The saline group was labeled with 113, 114, 115, METH group with 116, 117, 118 and the pool with 121. Samples from each group were combined together and subjected to peptide purification followed by OFFGEL fractionation.

### Peptide purification and OFFGEL fractionation

Labeled samples were subjected to cleanup using a Waters Oasis MCX cartridge (Waters, Milford, MA), a mixed mode cation exchange cartridge packed with materials containing both hydrophobic properties and negatively charged groups. 1 mL of 0.2% formic acid was added to each sample to obtain a final pH of 3. Each MCX cartridge was equilibrated by slowly passing 1 mL of 1∶1 methanol: water across the cartridge. The sample was applied at a flow rate of 1 drop per second followed by subsequent wash with 1 mL of 5% methanol, 0.1% formic acid followed by 1 mL of 100% methanol. The bound peptide was eluted with freshly prepared elution buffer (50 µl of 28% NH_4_OH, 950 µl of methanol), dried in a speed vac and stored at −80C until subjected to OFFGEL fractionation.

Peptide fractionation was performed using the 3100 OFFGEL Fractionator (Agilent Technologies, Santa Clara, CA) following the manufacturer's protocol. The device was set up for the 12 fractions separation by using 13-cm-long IPG gel strip with a linear pH gradient ranging at 3–10. 100 µg of peptide digest was resuspended with focusing buffer to a final volume of 1.8 mL. 150 µL of this sample was loaded in each of the 12 wells. The sample was focused using the recommended method for OFFGEL peptides 12 wells fractionation with a maximum current of 50 µA. The focusing was stopped after total voltage reached 50 kVh. During focusing, oil was added to the electrodes to prevent any evaporation effect. After focusing, 50 to 200 µl of sample was recovered for each well and transferred in individual micro tubes. For maximum recovery of the peptides, 200 µl of (49∶50∶1) water:methanol:formic acid was added to each well, incubated for 15 min without voltage, dried in a speed vac and resuspended with the corresponding sample. Corresponding peptides fractions were concentrated by vacuum centrifugation prior to spotting onto a MALDI plate followed by LC-MS/MS analysis.

### Tempo LC and MALDI-TOF/TOF

The Tempo LC MALDI robotic spotting system equipped with a C18 reversed phase capillary column (AB Sciex, Foster City, CA) was used to further fractionate peptides from OFFGEL fractions, followed by data acquisition using 4800 MALDI TOF/TOF (AB Sciex) as previously published [19]. Briefly, using an in-house packed C18 column to separate fractions by HPLC gradient, LC fractions were spotted onto MALDI 1232-spot format plates, with a spotting interval of 24 s and applying 2.8 kV plate voltage. Data was acquired from LC MALDI spot fractions using 4800 MALDI TOF/TOF equipped with a 200 Hz repetition rate Nd:YAG laser. Spectra from a total of 800 laser shots was accumulated for each TOF MS spectrum between 800 and 4000 *m*/*z*. Data-dependent MS/MS mode was operated using CID gas (air) and 2 kV collision energy. Programmed laser stop conditions were employed for the accumulation of MS/MS spectra from 800 to 4000 laser shots.

### Data Analysis

TOF MS and MS/MS spectra were analyzed using Protein Pilot v.2.0.1 software, which utilizes the Paragon scoring algorithm [20]. Search parameters included: iTRAQ 8plex (peptide labeled) sample type, iodoacetemide cys alkylation, trypsin digest, biological modifications ID focus and thorough search effort. The protein FASTA database used for searches was a concatenated “target-decoy” version of *Macaca* subset of the NCBInr database (20090731).

### Cell culture

Rat striatal neurons from embryonic day 18 Sprague Dawley rat pups were cultured in Neurobasal media containing B27 (Invitrogen, Carlsbad, CA). For western blot analysis, cells were plated at a density of 1×10^6^ per well onto a 6-well plate and 2.5×10^5^ cells per well for immunolabeling, in a 24-well plate containing coverslips. All treatments were performed after culturing 8 days *in vitro* (DIV).

### MTT assay

Cell viability was measured by 3-(4,5-dmethylthiazol-2-yl)-2,5-diphenyl Tetrazolium bromide (MTT) method. Rat striatal neurons were seeded in 96-well plates and exposed to various concentrations of METH for 24 h. Post treatment, MTT tetrazolium salt dissolved in Hank's balanced salt solution at a final concentration of 5 mg/ml was added to each well and incubated at 37°C till the formation of formazan crystals. The medium was carefully aspirated from each well and 200 µl of dimethyl sulfoxide was added to dissolve the formazan crystals and the absorbance of each well was obtained using a plate counter at test and reference wavelengths of 570 nm and 630 nm, respectively.

### Immunofluorescence

Prior to double labeling, both the control and METH treated rat striatal neurons were fixed in 4% paraformaldehyde. After three washes with PBS, cells were permeabilized with 0.25% Tween-20 in PBS for 20 min at room temperature and washed twice with PBS. Cells were incubated in 10% normal goat serum with 0.25% Tween-20 in PBS for 30 min at room temperature and then incubated with anti-ATP1A3 (1∶50 Santa Cruz) at 4°C overnight. After incubation with fluorescence-labeled secondary antibody (1∶100, donkey anti-goat IgG, Invitrogen) for 1 h at room temperature, the second primary antibody anti-MAP2 (1∶500, Sternberger Monoclonals, Lutherville, MD) was added at 4°C overnight. After incubation with second secondary antibody (1∶100, chicken anti-mouse IgG, Invitrogen), cells were washed and mounted with Prolong Gold with DAPI and analyzed by microscopy (Carl Zeiss, USA). For staining with Synaptophysin, cells earlier incubated with anti-ATP1A3 were incubated in 10% normal goat serum with 0.25% Tween-20 in PBS for 30 min at room temperature and then incubated with anti-SYP (1∶500 Santa Cruz) at 4°C overnight. Cells were washed and mounted with Prolong Gold with DAPI and analyzed by microscopy (Carl Zeiss, USA)

### Pharmacological inhibitors

The specific ERK1/2 inhibitor U0126, JNK inhibitor SP600125 and p38 inhibitor SB203580 were purchased from Sigma (St. Louis, MO). For cell signaling experiments, cells were pre-treated with 20 µM each with U1026, SP600125 and SB203580 for 1 h followed by TNF-α and IFN-γ (6 h) and/or METH treatment (15 min) and analyzed by western blot analysis.

### Western blot analysis

SDS-PAGE electrophoresis was performed using NuPAGE gel system (Invitrogen) in 6% Tris-Glycine (ATP1A3) and 4–12% gradient gels (pERK1/2 and pJNK) under reducing conditions. For western blot analyses, 10 µg of rat striatal cell lysate were loaded per lane. Electrophoresis followed by transfer and immunodetection was performed as previously described [21]. Nonspecific antibody binding was blocked using 5% nonfat dried milk for 1 hr at room temperature. Immunoblotting was carried out with antibodies against ATP1A3 (1∶2500, Santa Cruz Biotechnology, Santa Cruz, CA), phosphorylated ERK1/2 (1∶500, Cell Signaling Technology, Danvers, MA), phopshorylated JNK (1∶500, Cell Signaling Technology) followed by secondary antibody (1∶50,000 HRP conjugated anti goat IgG for ATP1A3 and 1∶5000 HRP conjugated anti rabbit for pERK1/2 and pJNK respectively; GE Healthcare, Little Chalfont, UK). Blots were developed with 1∶1 solution of Super Signal West Pico Chemiluminescent Substrate and Luminol/Enhancer (Thermo Fisher Scientific, Rockford, IL, USA).

### Statistics

Data represented are from three independent experiments using the tests described in the text and figure legends. Differences were considered significant at p<0.05. Tests were performed using Prism software (GraphPad Software Inc., San Diego, CA) for Macintosh.

## Results

This study comprised of six rhesus monkeys chronically infected with SIV with one group receiving chronic METH treatment mimicking human METH administration patterns both in frequency and treatment [13]. Since our goal was to examine the processes leading to CNS disease instead of the end-result of severe disease, animals were sacrificed prior to development of AIDS. To gain mechanistic clues associated with METH mediated CNS dysfunction, we employed a global quantitative mass spectrometry based proteomics approach by subjecting synaptosomal proteins isolated from the two groups on a LC-MALDI-TOF-MS/MS approach. A total of 890 proteins were identified (Table S1) of which 31 proteins were differentially expressed between the two groups. Since the differences observed in this study were subtle yet significant, we set a cutoff change of 1.2 as the cutoff. This criterion identified a total of eight proteins of which four were up regulated and four down regulated in the METH treated group (Table 1). Among the four up regulated proteins, we identified two transporters (glial affinity glutamate transporter +1.28 fold) and sodium/potassium/calcium exchanger +1.28 fold) and two isoforms of the Na^+^/K^+^ ATPase (NKA) family- alpha 3 subunit (+1.21 fold) and beta 1 subunit (+1.28 fold). Amongst the down-regulated proteins identified included enzymes aldehyde dehydrogenase (−1.31 fold), phosphoinositide-specific phospholipase C beta 1 isoform (−1.23 fold), Moesin (−1.26 fold) and the immunoglobulin heavy chain V (−1.2 fold).

**Table 1:** Synaptic proteins differentially regulated between the two groups of monkeys.

Protein identified	Fold change	p-value
Solute carrier family 1 (glial high affinity glutamate transporter), member 3 isoform 9	+1.28	0.014
Solute carrier family 24 (sodium/potassium/calcium exchanger), member 2 isoform 1	+1.28	0.038
Na^+^/K^+^ -ATPase beta 1 subunit isoform 4	+1.25	0.02
Na^+^/K^+^ -ATPase alpha 3 subunit 1	+1.21	0.01
Aldehyde dehydrogenase 1A1 isoform 2	−1.31	0.015
Moesin	−1.26	0.013
Similar to phosphoinositide-specific phospholipase C beta 1 isoform a	−1.23	0.049
Similar to Ig heavy chain V-II region SESS precursor	−1.2	0.002

Unpaired student t-test was used to determine the significance.

**Figure 1:**
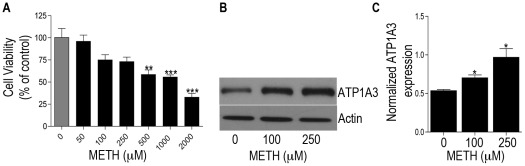
MTT assay showing viability of cells post treatment with varying amounts of METH for 24 h. **p<0.01, ***p<0.001 (A). (B) Representative western blot confirming up regulation of ATP1A3 in rat striatal neurons treated with indicated concentrations of METH for 24 h as compared to controls. (C) Bar graphs showing significant increase in ATP1A3 expression post METH treatment versus control. Data represented as Mean ± SEM of three independent experiments. *p<0.05.

**Figure 2:**
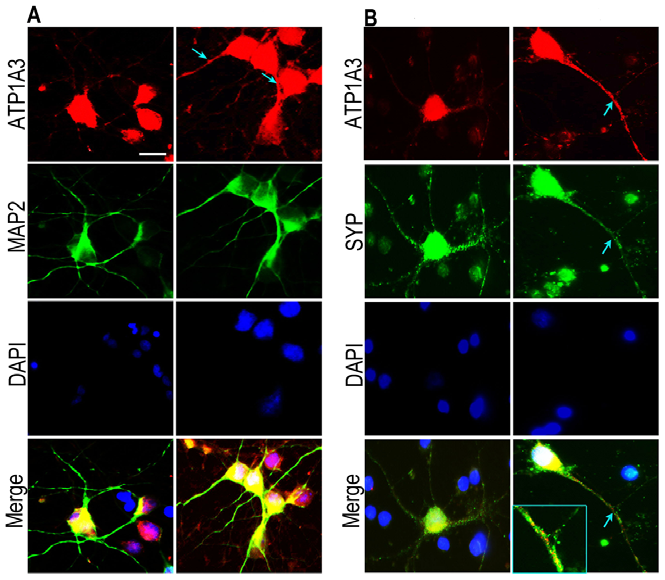
Primary rat striatal neurons stained for levels and distribution of ATP1A3 after 250 µM METH treatment for 24 h along with MAP2 staining for neuronal structure. (A) and with synaptic marker synaptophysin (B). DAPI was used to stain cell nucleus. Cells treated with METH show an increase and redistribution of ATP1A3 along the neuronal processes indicated by arrowheads and in the nerve terminals (inset). Scale bar = 10 µm.

The identification of Na^+^/K^+^ ATPase alpha 3 subunit (ATP1A3) which was up regulated in the METH treated group was intriguing given its role in maintaining neuronal homeostasis and its neuronal localization. The NKA utilize >50% of the total cell's energy and the increase seen in our study could lead to a substantial difference in energy utilization and thus can impact neural homeostasis. In order to examine whether METH itself can increase the expression of ATP1A3 at the protein level, we isolated and cultured primary rat striatal neurons and subjected them to METH treatment. While numerous animal and *in vitro* studies utilize a high concentration, neurotoxic dosing of METH, we wanted to mimic a more physiological concentration where neuronal death is not prominent acutely. We first assessed neuronal cell viability over broad range of METH concentrations. Results from MTT assay showed that concentrations of 500 µM or greater were toxic (**p<0.01 for 500 µM and ***p<0.001 for 1000 and 2000 µM) and thus a dose of 250 µM was used for all subsequent experiments (Figure 1A). This concentration is in the range documented from other published clinical studies that have measured METH levels in blood, urine or tissue samples of METH abusers [22]–[28]. Next, we found that 250 µM METH treatment of rat striatal neurons led to a significant increase in ATP1A3 expression. This was demonstrated both by western blot analysis (Figure 1B) and immunofluorescence, which revealed an increase in ATP1A3 expression in neurons after METH treatment, evident in both the soma and neuronal processes (Figures 2A and 2B). Thus not only does METH treatment *in vivo* lead to increased ATP1A3 expression, METH treatment *ex vivo* reveals a direct distinct response in the neurons through up regulation of ATP1A3.

**Figure 3:**
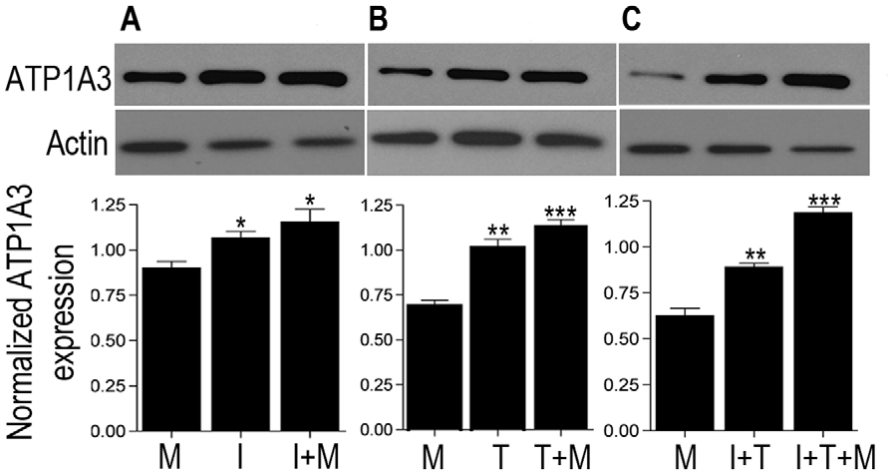
Western blot analysis on rat striatal neurons pre-treated with indicated cytokines (10 ng each) for 6 h followed by METH treatment for 24 h showing increased ATP1A3 expression compared to METH only and cytokine only treatments. (M – METH, I – Interferon-γ, T – TNF-α). Bar graphs showing significant increase in ATP1A3 expression post treatment with the cytokines. Data represented as Mean ± SEM of three independent experiments. *p<0.05, **p<0.01, ***p<0.001 versus METH treatment.

We next examined the potential role of brain HIV infection, which in conjunction with METH leads to neuronal damage. Though HIV does not infect the neurons, the ensuing damage to neurons and their subsequent degeneration, is via macrophages [29], microglia [30], [31] and astrocytes [32], [33] with the subsequent release of viral proteins and proinflammatory cytokines. Studies from other groups including ours have documented a role for the increased expression of proinflammatory cytokines, TNF-α and IFN-γ in CNS tissues during HIV infection and associated pathophysiology of HAND [34]–[36]. To recapitulate the ensuing cascade of neuronal damage occurring in the brain *in vitro*, we treated rat striatal neurons with proinflammatory cytokines TNF-α and IFN-γ followed by METH treatment for 24 h. Western blot analysis revealed an increase of ATP1A3 expression and the expression was further augmented with METH treatment when neurons were treated with a combination of TNF-α and IFN-γ (Figure 3). Taken together these data point towards a potential involvement of the virus-induced inflammation in the brain, which in presence of METH leads to further increase in the expression of ATP1A3.

**Figure 4:**
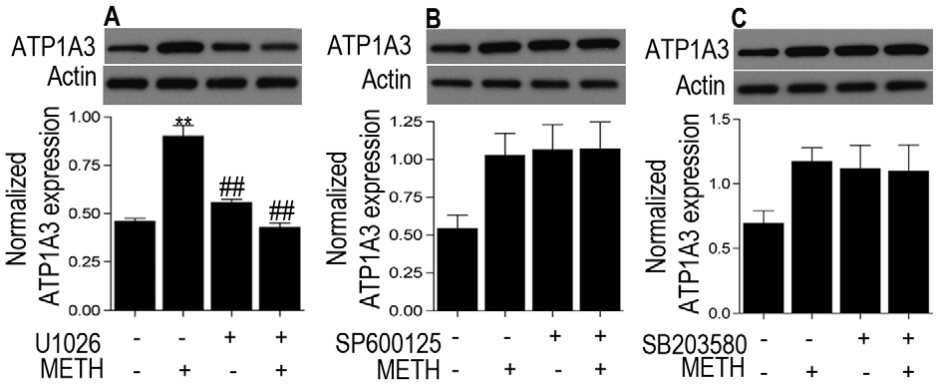
Western blot analysis on rat striatal neurons pre-treated with ERK1/2 JNK and p38 inhibitors U1026, SP600125, and SB203580 respectively followed by METH treatment shows U1026 blocks the METH-induced increase in ATP1A3 expression. (A) but SP600125 or SB203580 do not (B, C). Data represented as Mean ± SEM of three independent experiments. **p<0.01 versus control, ##p<0.01 versus METH treatment.

**Figure 5:**
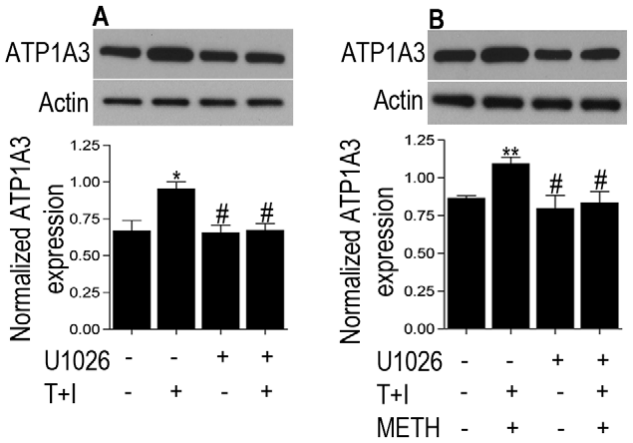
Western blot analysis on rat striatal neurons pre-treated with the ERK inhibitor U1026 followed by cytokines treatment (6 h) in absence (A) and presence (B) of METH treatment for 15 min show abrogation of the increase in ATP1A3 expression. Data represented as Mean ± SEM of three independent experiments. *p<0.05, **p<0.01 versus control, #p<0.05 versus cytokine ± METH treatment.

Emerging studies have documented the role of ATP1A3 as a signaling molecule involving the mitogen activated protein kinase (MAPK) family [37]. Also, both METH and HIV have been shown to activate members of the mitogen activated protein kinase (MAPK) family including the extracellularly regulated kinase 1/2 (ERK1/2), c-Jun N-terminal kinase (JNK) and p38 MAPK. Western blot analysis of lysates from METH treated rat striatal neurons showed a time-dependent activation of both pERK1/2 and pJNK with a peak expression at 15 min post treatment with METH (Figure S1, p<0.01 for pERK1/2 and p<0.05 for pJNK versus control). To further confirm involvement of the specific pathways in the induction of ATP1A3, neurons were pre-treated for 1 h with specific pharmacological inhibitors: U1026 for ERK1/2, SP600125 for JNK and SB203580 for p38, followed by METH treatment for 15 min. The increase in expression of ATP1A3 was significantly abrogated with pretreatment with U1026 (p<0.01 versus METH treated cells) but not SP600325 or SB203580, thus implicating the involvement of the ERK1/2 pathway (Figure 4), which was indeed activated by METH (Figure S1). Having ascertained the implication of the ERK1/2 pathway, we hypothesized if both the virus and drug induced expression of ATP1A3 can be attenuated using the ERK1/2 inhibitor U1026. Pretreatment of neurons for 1 h with U1026 followed by cytokines treatment for 6 h and later in presence and absence of METH treatment for 15 min revealed a significant block in the cytokine ± METH induced increase in ATP1A3 expression (Figure 5; p<0.05 versus cytokine+METH treated group). Together, these data implicate a role of ATP1A3 in HIV/METH induced CNS dysfunction and that this involves the ERK1/2 pathway.

## Discussion

In this study, we hypothesized that a quantitative iTRAQ based proteomics approach would allow us to identify differential protein fingerprints in the brain reflecting the effect of METH treatment in SIV infected monkeys, modeling HIV infection of humans and drug abuse. Profiling of synaptosomes isolated from the caudate nucleus of the two groups of monkeys identified several synaptic proteins which were differentially expressed, of which we focused on the neuron specific Na/K-ATPase alpha 3 subunit (ATP1A3), which was up regulated after METH treatment. *In vitro* studies confirmed that it is up regulated by METH as well as proinflammatory cytokines typically produced during HIV infection in the brain.

ATP1A3, which is a critical component of the Na/K-ATPase complex carries out active transport of sodium and potassium across the cell membrane and maintains the chemical gradients of these ions. These gradients are of fundamental importance and are crucial in regulating a plethora of cellular functions including regulation of cell volume, osmotic activity, transport of neurotransmitters across the plasma membrane and excitability of neuronal cells [38]. The *in vivo* effects of reductions in ATP1A3 have been studied in humans and mice. Genetic studies have identified that mutations in ATP1A3 are linked with rapid-onset dystonia Parkinsonism in humans [39]. In mouse mutagenesis studies, a dysfunctional allele of ATP1A3 was produced that was lethal when homozygous due to epileptic seizures [40]. Hetereozygous mutant mice showed hyperactivity, spatial learning and memory deficits, and increased locomotion induced by METH treatment [41]. It is hypothesized that a decrease in ATPase activity leads to pronounced levels of ATP availability and rendering the animals hyperactive. However in our case, over expression of ATP1A3 is found, pointing to a higher consumption of ATP, potentially impacting energy homeostasis in individuals who are HIV+ and use METH. Though the fold change in ATP1A3 expression seen between the groups is subtle, at the synaptic level, given the large role NKA play in neuronal energetics such subtle alterations could impact dynamics of neurotransmission.

The synapse is a key functional unit in the brain important in regulating neurotransmission and thus it is plausible that both HIV and METH could impact the dynamics of neurotransmission. An earlier study examined synaptosomal proteins from the neocortex of HIV-infected decedents with AIDS and HIV encephalitis. In this study, an increased expression of immunoproteasome subunits was found that was inversely related with the brain HIV viral load [42]. Clearly further examination of synapses, key in neuronal function, can continue to provide important information regarding the effects of HIV, drugs of abuse, and other neurological disorders.

Our study has revealed that METH acts through MAPKs to increase ATP1A3 expression. Interestingly recent studies have identified ATP1A3 as a significant signal transducing molecule itself, signaling through MAPKs [37]. Activation of the MAPK ERK1/2 has mostly been linked to cell survival, proliferation and differentiation given their activation by mitogens and some cell survival factors [43]–[45]. However, activation of ERKs has also been implicated in neuronal death in certain *in vitro* models of neurotoxicity [46]–[49]. Thus the possibility exists that a feedback loop exists, in which METH signaling through MAPKs increase ATP1A3, which then itself helps further increase MAPK signaling.

Neurons are highly specialized and polarized cells with large energy dependency that consume vast amounts of ATP. Importantly, neuronal transmission via release of neurotransmitters into the synaptic cleft is ATP dependent. Central to this regulation is the Na^+^/K^+^-ATPase that maintains the intracellular homeostasis with a tight regulation of Na^+^ and K^+^ ions. Imbalances in the intracellular currents especially sodium currents could severely impact the ion gradient and subsequent effect on ion channels. There is little yet emerging evidence on alterations in brain energy metabolism in HIV patients and who are dependent on METH. An earlier study employing proton magnetic resonance spectroscopy showed a decrease in the levels of the brain metabolites N-acetyl aspartate and an increase in myo-inositol levels in the brains of HIV+ individuals with a chronic history of METH abuse [50]. In another study, METH exposure resulted in mitochondrial oxidative damage and caused dysfunction of primary human T cells [51]. A more recent study demonstrated that METH exposure impairs glucose uptake and metabolism in human neurons and astrocytes suggesting that deprivation of glucose-derived energy may contribute to neurotoxicity of METH abusers [52]. It is thus reasonable to hypothesize that alterations in energy metabolism induced by METH could further be augmented in synergy with the virus and thereby leading to neurodegeneration.

Given the synaptic localization of ATP1A3 and its role in maintaining neuronal homeostasis, an increase in its expression by METH treatment could potentially impact the dynamics of neurotransmitter release. Further *in vitro* and *in vivo* studies in model systems would allow the examination of this possibility.

## Supporting Information

Figure S1 **A time course increase in phosphorylated forms of ERK1/2 (A) and JNK (B) in rat striatal neurons treated with METH.** Data represented as Mean ± SEM of three independent experiments. *p<0.05, **p<0.01 versus control.
(TIF)

Table S1 **Complete list of synaptic proteins identified between the two groups of monkeys. Unpaired student t-test was used to determine the significance.**
(XLS)
