# Supplementary figures and images for: Methamphetamine and Inflammatory Cytokines Increase Neuronal Na+/K+-ATPase Isoform 3: Relevance for HIV Associated Neurocognitive Disorders
Source: PLoS One. 2012 May 25;7(5):e37604. doi: 10.1371/journal.pone.0037604 (PMC3360751; doi:10.1371/journal.pone.0037604)

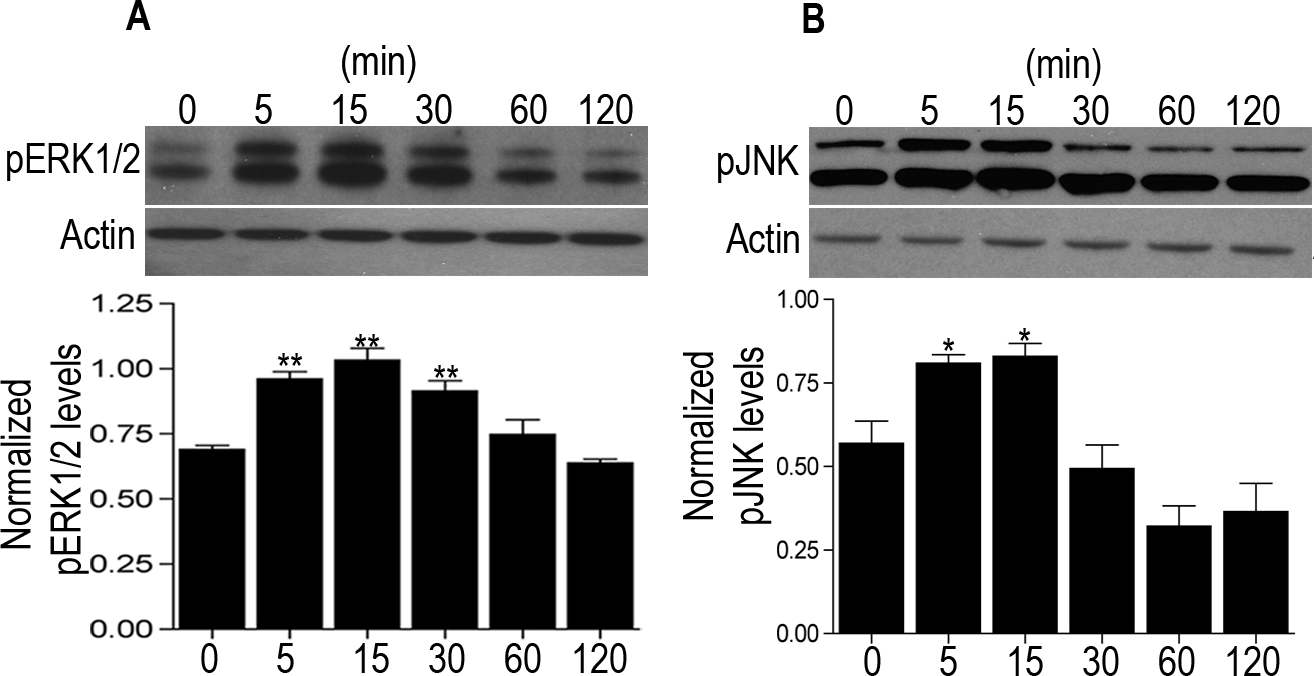

Supplement: Figure S1 — A time course increase in phosphorylated forms of ERK1/2 (A) and JNK (B) in rat striatal neurons treated with METH. Data represented as Mean ± SEM of three independent experiments. *p<0.05, **p<0.01 versus control. (TIF) [file pone.0037604.s001.tif]
